# Women's knowledge and perceptions of malaria and use of malaria vector control interventions in Kersa, eastern Ethiopia

**DOI:** 10.3402/gha.v6i0.20461

**Published:** 2013-05-23

**Authors:** Tesfaye Gobena, Yemane Berhane, Alemayehu Worku

**Affiliations:** 1 College of Health and Medical Science, Haramaya University, Harar, Ethiopia; 2 Addis Continental Institute of Public Health, Addis Ababa, Ethiopia; 3 School of Public Health, Addis Ababa University, Addis Ababa, Ethiopia

**Keywords:** IRS, LLINs, replastering of walls, LLIN use, women's knowledge and perceptions of malaria, Ethiopia

## Abstract

**Background:**

Ethiopia has a long history of controlling malaria using vector control tools. Community knowledge and perceptions of malaria and use of malaria vector control interventions vary.

**Objective:**

The aim of this study was to determine malaria-related knowledge and perceptions among women and to determine the use of malaria vector control interventions, mainly indoor residual spraying (IRS) and insecticide-treated nets (ITNs), among households in Kersa, Eastern Ethiopia.

**Design:**

A cross-sectional survey was conducted in Kersa Demographic Surveillance and Health Research Center (KDS-HRC) site from October to November 2010. A total of 2,867 households were involved in the study. The data was collected via face-to-face interviews with the women of the household using a pre-tested questionnaire. The questionnaire contained closed, semiclosed, and open-ended questions to explore the reasons for non-use of the interventions. Each knowledge, perception, and practice question was analyzed separately.

**Results:**

Of the total women, 2,463 (85.9%) had heard of malaria. Of them, 1,413 (57.4%) mentioned malaria as a communicable disease. But, only 793 (56.1%) of them associated mosquito bites with malaria transmission. Seven hundred and ninety-eight of the respondents (27.8%) had IRS coverage, and of these, 59 (7.4%) had re-plastered their interior walls following the application of insecticides. Of net-owning households, 33.5% had used at least one long-lasting insecticide-treated net (LLIN) the night before the survey. Societal reasons such as holy days and dislike of the insecticide mainly due to fear of its effects on their livestock, were the main reasons for re-spondents replastering their walls.

**Conclusions:**

A substantial number of women had heard about malaria, but there was a knowledge gap regarding the route of malaria transmission. Less than one-third of the surveyed household houses were sprayed with insecticides, and a low proportion of net-owning households actually used their nets. Efforts must be made to ensure the correct channeling of information about malaria, particularly regarding the importance of using malaria vector control interventions. Furthermore, to maximize the benefit of the intervention in the district, IRC coverage and LLIN use need to be stronger.

Ethiopia is one of the many sub-Saharan African countries that is seriously affected by malaria (1). About three-fourths of the total area and two-thirds of the population are at risk of this infection. The transmission of malaria in Ethiopia is seasonal and unstable (2). In recent years, a substantial decrease in cases has been reported in the country, although it continues to be a major public health problem (3).

Vector control using insecticide-treated nets (ITNs) and indoor residual spraying (IRS) is most effective in the prevention of malaria transmission (4). Empirical evidence demonstrates the effectiveness of ITNs in reducing instances of malaria infection (5, 6). IRS is another highly effective tool for obtaining rapid, large-scale impacts on both vector populations and malaria morbidity and mortality (7). The effectiveness of IRS in reducing malaria transmission and disease burden was demonstrated in southern African countries (7, 8) and India (9). Furthermore, community-wide benefits of IRS have been reported in Kenya (10). Recently, a study in Ethiopia demonstrated the impact of IRS with dichloro-diphenyl-trichloroethane (DDT) in reducing the incidence of malaria (11).

ITNs and IRS are the most common malaria vector control tools in Ethiopia (12), along with mosquito larval source reduction (12). The use of IRS has a long history in Ethiopia (13). IRS insecticides commonly used in Ethiopia include DDT, malathion, and deltamethrin. Due to the resistance of malaria vectors to DDT, the use of this insecticide for IRS was discontinued in 2009, and deltamethrin is currently being used as an interim substitute in IRS operations (14).

The World Health Organization (WHO) recommends that national governments introduce and/or scale up coverage of targeted IRS as a primary malaria control intervention strategy to achieve malaria targets (15). In Ethiopia, IRS coverage has increased from 20% in 2007 (16) to 46.6% in 2011 (17). In the same period, it increased from 12.5 to 43% in the Oromia Regional State. But, overall the country planned to increase IRS in malaria-epidemic-prone localities to 60% in 2010 (18).

The success of malaria control interventions requires their high coverage and utilization at community and individual levels (15, 19). One of the challenges for the appropriate use of these interventions is inadequate knowledge and perceptions of malaria at the community level. Previous studies have showed varied levels of malaria-related knowledge in Ethiopia. For example, a study reported that 93% of the participants knew that malaria could be transmitted through mosquito bites (20); another study, in the Wonago district of Ethiopia, reported that 42.3% of the respondents described mosquitoes as the main transmission mechanism for malaria (21); and the 2007 nationwide survey reported that 35.8% of the respondents mentioned mosquito bites as the cause of malaria (22). To achieve universal coverage of malaria vector control interventions, it is imperative to understand communities’ malaria-related knowledge and perceptions and their use of available interventions at different eco-epidemiological strata of the country. The aim of this study was to assess the level of women's knowledge and perceptions related to malaria and the use of malaria vector control interventions in semirural communities.

## Methods

### Study setting

The study was conducted in Kersa Demographic Surveillance and Health Research Center (KDS-HRC) site of Kersa district, eastern Ethiopia, during the high malaria transmission season of October–November 2010 (2). The district is recognized as a malaria endemic and fringe zone (23, 24). In public health facilities of the district, a total of 2,032 clinical and 340 confirmed malaria cases were reported in 2010–2011. Of the total confirmed cases, 79.7% were *Plasmodium falciparum*, 17.6% were *Plasmodium vivax*, and the rest 2.6% were a mix of *P. falciparum* and *P. vivax* (unpublished observation).

The KDS-HRC site includes 12 randomly selected *kebeles* of the district (a *kebele* is the smallest administrative unit in Ethiopia). These *kebeles* were selected based on a mix of altitudes and the urban–rural composition of the district. Nine of the 12 *kebeles* were in the IRS program as targeted *kebeles* (District Health Office, November 15, 2009, personal communication.

### Sample size and sampling

This study was conducted as part of a cross-sectional survey of the coverage and use of malaria interventions. A total of 2,912 households were targeted to take part in the survey. The sample size estimation is discussed in Reference (25). The study sample was taken from the KDS-HRC database, and it is proportional to the size of households of each *kebele* using simple random sampling. The surveillance database has a unique identification number for each household under surveillance.

### Data collection

This survey was conducted during the main malaria transmission season following the main rainy season. A total of 12 experienced and trained female data collectors and three nurse supervisors were involved in data collection and field supervision, respectively. The data collection instrument and techniques are described in Reference (25). The questionnaire contained questions about such topics as knowledge of malaria; its cause, symptoms, prevention, and curability; and the use of malaria interventions. The questions contained closed ended, semi-closed ended, and open ended questions. The open-ended questions were used to explore the reasons why some households did not spray and why some others replastered their walls following the application of insecticide, and the perceptions of women on the cause of malaria, malaria transmission, and malaria intervention methods used for prevention. The purpose of the study was explained to the women and/or to the head of the household, and their informed verbal consent was obtained before the questionnaire was administered. The data collectors gave advice to household members who manifested signs and symptoms of malaria during the survey to visit the nearby health facility for diagnosis and treatment. In addition, advice was also given to the women or head of the households on the relevance of proper use of malaria control interventions.

### Data analysis

The data were double-entered into EpiData 3.1 by two data operators. Next, the data were exported into SPSS 16.0 software, then cleaned and analyzed using the same software. IRS was calculated as the percentage of households that sprayed in the 12 months before the survey. Replastering or repainting of a household's walls was calculated as the proportion of household walls that had been replastered or repainted following the application of insecticide. Knowledge of malaria cause, transmission, prevention, and severity was computed as the proportion of women who correctly answered each of the specific questions regarding these topics. An unadjusted odds ratio (OR) was calculated between malaria-related information and the use of malaria vector control interventions. A crude OR was computed with 95% confidence intervals (CI) to identify factors associated with non-spraying of insecticide. A Pearson chi-square test was used to test the significance difference of proportions in the categorical variables. *P* value≤0.05 was taken as a cutoff point for statistical significance. The open-ended questions’ information on non-spraying and on replastering of walls was coded based on the identified thematic areas.

## Results

### The characteristics of the study subjects

Of the targeted 2,912 households, 2,867 (98.5%) were involved in the study. Thus, a total of 2,867 women were interviewed. The mean age of the respondents was 34.6±12.3 years and ranged from 15 to 93 years. A majority of the respondents, 2,486 (86.7%), did not have formal education. A majority of the surveyed households (2,635; 91.9%) were residing at altitudes lower than 2,500 m above sea level, and the rest (232; 8.1%) resided at altitudes above 2,500 m.

### Women's knowledge and perceptions of malaria

Of the women who were interviewed, 2,463 (85.9%) had heard of malaria. The respondents heard about malaria from more than one source of information. Radio (1,064; 37.1%), health institutions and health professionals (946; 33.0%), friends (749; 26.1%), and household members (719; 25.1%) were the main sources of information on malaria. A larger proportion of women living at altitudes lower than 2,000 m, or the lowlands (96.4%); women in urban settings (96.2%); women who had the highest level of formal education (94.5%); and women in radio-owning households (88.3%) had heard of malaria compared to their counterparts (Table 1).

**Table 1:** Proportion of women who had heard of malaria, by some sociodemographic and climatic characteristics of Kersa district, 2010

Variables (*n*=2,867)	*n*	Frequency (%)
Age category of women		
15–29 years	1,092	918 (84.1)
30–44 years	1,130	989 (87.5)
≥45 years	645	556 (86.2)
Level of education of women		
Illiterate (no formal education)	2,486	2,104 (84.6)
Grades 1 to 4	182	171 (94.0)
Grades≥5	199	188 (94.5)
Radio possession of household		
Yes	1,146	1,012 (88.3)
No	1,721	1,451 (84.3)
Place of residence		
Urban	341	328 (96.2)
Rural	2,526	2,135 (84.5)
Altitude above sea level		
≤2,000 m	535	516 (96.4)
>2,000 m	2,332	1,947 (83.5)

Of those women who had heard of malaria (2,463), 1,413 (57.4%) reported malaria as a communicable disease. But, only 793 (56.1%) of the women mentioned mosquito bites as the route of malaria transmission. A larger proportion of women living in altitudes lower than 2,000 m (71.2%); women in rural areas (60.5%); literate women, with an education of grade 5 or above (65.4%); and women in the age category of 30–44 knew that malaria is transmittable compared to their counterparts. Of those who heard about malaria, 1,954 (79.3%) reported malaria as a preventable disease. Most of them mentioned more than one means of malaria prevention. Accordingly, 1,606 (82.2%), 1,020 (52.2%), and 559 (28.6%) mentioned mosquito nets, IRS, and fumigation with local herbs as malaria prevention tools, respectively. A larger proportion of women in the 30–44 age category (82.9%), those with the highest education level (84.8%), rural residents (83.6%), and residents in altitudes lower than 2,000 m knew that malaria is a preventable disease in comparison with their counterparts (Table 2). A majority of them, 2,423 (98.4%) and 2,322 (94.3%), mentioned malaria as a severe disease and a curable disease, respectively.

**Table 2:** Women's knowledge of malaria transmission and its preventability, by some sociodemographic environmental factors of Kersa district, 2010

Variables (*n*=2463)	*n*	Communicable frequency (%)	Preventable frequency (%)
Age category of women			
15–29 years	922	485 (52.6)	722 (78.1)
30–44 years	984	624 (63.4)	812 (82.9)
≥45 years	557	304 (54.6)	420 (75.1)
Level of education of women			
Illiterate (no formal education)	2,105	1,202 (57.1)	1,675 (79.4)
Grades 1 to 4	170	88 (51.8)	123 (72.4)
Grades≥5	188	123 (65.4)	156 (84.8)
Radio possession of household			
Yes	1,014	547 (53.9)	782 (76.9)
No	1,449	866 (59.8)	1,172 (81.1)
Place of residence			
Urban	326	121 (37.1)	168 (51.4)
Rural	2,137	1,292 (60.5)	1,786 (83.6)
Altitude above sea level			
≤2,000 m	514	366 (71.2)	420 (81.2)
>2,000 m	1,949	1,047 (53.7)	1,534 (78.8)

### Utilization and factors related to the interventions

Of the total surveyed households, 798 (27.8%) houses had been sprayed with insecticide in the 12 months preceding the survey. Of these, 59 (7.4%) got their walls replastered following application of the insecticide. A total of 1,879 (65.5%) households had at least one LLIN, and of these, 630 (33.5%) used at least one LLIN the night before the survey. Women in the households who had reasonable knowledge on the transmission and prevention of malaria used the interventions significantly more than their counterparts (Table 3).

**Table 3:** Use of malaria interventions, by knowledge of malaria and its transmission, prevention, and severity, in Kersa, eastern Ethiopia, 2010

Characteristics	IRS (*n*=798) No (%)	Replastered (*n*=59) No (%)	Owns at least one LLIN (*n*=1,879) No (%)	Uses at least one LLIN (*n*=630) No (%)	Chi-square test (*P* value)
Heard about malaria (*n*=2,867)					
Yes	670 (84.0)	56 (94.9)	1,559 (83.0)	534 (84.8)	3.12 (0.08)
No	128 (16.0)	3 (5.1)	320 (17.0)	96 (15.2)	
Malaria is a communicable disease (*n*=2,463)					
Yes	464 (58.1)	35 (59.3)	1,064 (56.6)	416 (66.0)	35.20 (0.0001)
No	191 (23.9)	18 (30.5)	448 (23.8)	107 (16.9)	
I do not know	11 (14)	3 (5.1)	39 (2.1)	9 (1.4)	
Route of transmission of malaria (*n*=1,413)					
Mosquito bite	365 (46.0)	26 (44.1)	668 (35.6)	339 (53.8)	99.80 (0.0001)
Others	97 (12.2)	9 (15.3)	400 (21.3)	79 (12.5)	
Malaria is a preventable disease (*n*=2,463)					
Yes	591 (74.1)	47 (79.7)	1,296 (69.0)	490 (77.8)	45.18 (0.0001)
No	73 (9.1)	8 (13.6)	193 (10.3)	39 (6.2)	
I don't know	7 (0.9)	1 (1.7)	73 (3.9)	6 (1.0)	
Malaria is a severe disease (*n*=2,463)					
Yes	665 (83.3)	55 (93.2)	1,540 (82.0)	527 (83.7)	Not valid
No	4 (0.5)	0 (0.0)	11 (0.6)	5 (0.8)	
I don't know	1 (0.1)	1 (1.7)	2 (0.1)	2 (0.3)	
Malaria is a curable disease (*n*=2,463)					
Yes	669 (83.4)	56 (94.9)	1,472 (78.3)	526 (83.5)	Not valid
No	2 (0.3)	0 (0.0)	79 (4.2)	5 (0.8)	
I don't know	0 (0.0)	0 (0.0)	1 (0.02)	1 (0.2)	

IRS coverage in lowland households (crude OR=13.85, 95% CI: 11.10, 17.29), LLIN-owning households (crude OR=3.41, 95% CI: 2.78, 4.18), LLIN-using households (crude OR=5.56, 95% CI: 4.58, 6.75), and households of women who knew that malaria is preventable (crude OR=1.29, 95% CI: 1.04, 1.60) was significantly higher than that of their counterparts. Households with mud-type houses and radio-owning households were sprayed with insecticide less often than those of their counterparts (Table 4) .

**Table 4:** Association of household characteristics with IRS spraying in households of the Kersa district, 2010

	IRS sprayed			
			
Variables (*n*=2867)	Yes No (%)	No No (%)	Crude OR (95%CI)	*P*
Wall types				
Mud and earth	410 (51.4)	1,647 (79.6)	0.27 (0.23, 0.32)	0.001
Wood, cement, and others	388 (48.6)	422 (20.4)	1	
Roof types				
CIS	648 (81.2)	1,624 (78.5)	1.18 (0.96, 1.46)	0.11
TR and others	150 (18.8)	445 (21.5)	1	
Radio possession				
Yes	262 (32.8)	884 (42.7)	0.66 (0.55, 0.78)	0.001
No	536 (67.2)	1,185 (57.3)	1	
Altitude above sea level				
≤2,000 m	397 (49.7)	138 (6.7)	13.85 (11.10, 17.29)	0.001
>2,000 m	401 (50.3)	1,931 (93.3)	1	
At least one LLIN owned				
Yes	662 (83.0)	1,217 (58.8)	3.41 (2.78, 4.18)	0.001
No	136 (17.0)	852 (41.2)	1	
At least one LLIN used				
Yes	363 (45.5)	270 (13.0)	5.56 (4.58, 6.75)	0.001
No	435 (54.5)	1,799 (87.0)	1	
Heard about malaria				
Yes	669 (83.8)	1,793 (86.7)	0.80 (0.63, 1.01)	0.06
No	129 (16.2)	276 (13.3)	1	
Malaria preventable				
Yes	591 (74.1)	1,363 (65.9)	1.29 (1.04, 1.60)	0.02
No	73 (9.1)	308 (14.9)	0.70 (0.51, 0.97)	0.03
I do not know	134 (16.8)	398 (19.2)	1	

### Reasons for not spraying and for replastering of walls

The reasons for not spraying and for replastering one's walls were assessed by using semiclosed and open-ended questions. The main reasons for not spraying certain houses among IRS target *kebeles* were as follows: the spray team never visited the household during the spray period (61.4%), a few of the households did not allow the spray team to spray their houses due to their dislike for insecticide and the fear that it may kill their livestock and honeybees (31.1%), the head of the household was not around the vicinity or elder household members were not at home when the spray team visited (3.2%), and other reasons (1.6%). Also, some households replastered their interior walls following the application of insecticide. The three main reasons for replastering were societal reasons such as holy days (57.6%) and New Year (20.3%), fear that chemicals might kill their livestock and honeybees (13.6%), and other reasons (8.5%).

## Discussion

This study revealed that the majority of women had heard of malaria, but a large proportion of them did not correctly associate malaria with mosquito bites. Radio programs, health facilities and health professionals, friends, and household members were the main sources of malaria information for these women. Along with low IRS coverage and LLIN use in the area, some households replastered their walls following the application of insecticide or before the residual efficacy period of the insecticide was over, which could decrease the actual IRS coverage to a lower level than that reported. Thus, the majority of the surveyed household residents did not obtain the maximum benefit of the interventions.

In this survey, there could be a potential bias in measuring IRS coverage in the 12 months preceding the survey and recalling the exact date of replastering the interior walls following the application of insecticide. The former was not a problem to ensure; the health extension workers knew the date of the previous spray of each of the *kebeles*. But, there might be a recall bias with regard to the exact date of the replastering.

Although women's awareness of the role of mosquitoes in malaria transmission is very crucial for the consistent and proper use of the available malaria prevention tools at the household level, nearly half of the women among those who had heard of malaria did not know the role of mosquito bites in malaria transmission. Although there is time variation, this study showed a higher proportion than the 2007 MIS study in the country, which reported that 74.6% of the respondents had heard of malaria, and of them 35.8% knew that mosquito bites are a cause of malaria (16). Another study in the country reported that 42.3% of the respondents mentioned mosquito bites as the main transmission mechanism for malaria (21). This was lower than a report from South Africa, which stated that 93% of the respondents had heard of malaria, and 84.6% of them correctly associated malaria with mosquito bites (26). Thus, the study showed a malaria-related knowledge gap among these semirural women despite international, national, and local efforts.

There is empirical evidence that IRS is effective at higher coverage in reducing mosquito density and consequently malaria morbidity and mortality (8); however, the study showed that fewer than one-third of the surveyed households’ dwellings were sprayed with insecticide. Although the IRS coverage in this study was higher than that of the Oromia Regional State, which reported 18.6% (27), it was far from the national targets (12). This is due to the fact that some *kebeles* in the district were not fully or partially included in the IRS program. Furthermore, some households in the targeted localities of the IRS program were not sprayed. This may be due to some operational problem in addressing all of the houses in the targeted localities of the IRS program. Although the proportion is small, some sprayed walls were replastered following the application of the insecticide, mainly due to social reasons and fear of the chemical, which decreased the actual effectiveness of IRS coverage of the study area.

Furthermore, the women's knowledge about malaria was not translated into LLIN use, although compliance with its use is more important to the control of malaria (28). Of LLIN-owning households, only 33.5% used at least one LLIN in the preceding night of the survey. It was lower than the 2007 national survey report (72.1%) (22) and a survey in Oromia and Amhara Regional States, which reported 65% usage (29), but it was consistent with another survey report from Ethiopia (30). This variability may be due to differences in eco-epidemiological strata in Ethiopia, correct knowledge regarding the route of malaria transmission, perceptions of malaria and ITN use, and access to malaria-related health services.

## Conclusions

A substantial number of eastern Ethiopian women have heard of malaria. But a large proportion of them did not make the correct association between malaria and mosquito bites. A majority of the surveyed households were not sprayed and did not use available mosquito nets. Thus, to maximize the benefit of the interventions, correct knowledge of the disease and tailored behavioral change communications are needed. Efforts must continue to properly channelize information about malaria transmission and the relevance of ITN and IRS use in malaria control. Further efforts must be made in increasing IRS coverage in the target *kebeles* of the district to maximize the benefit of the intervention.
